# Phase behavior of binary and ternary fluoropolymer (PVDF-HFP) solutions for single-ion conductors

**DOI:** 10.1039/d2ra04158h

**Published:** 2022-08-02

**Authors:** Jung Yong Kim

**Affiliations:** Department of Materials Science and Engineering, Adama Science and Technology University P. O. Box 1888 Adama Ethiopia; Center of Advanced Materials Science and Engineering, Adama Science and Technology University P. O. Box 1888 Adama Ethiopia jungyong.kim@astu.edu.et

## Abstract

A fluoropolymer poly(vinylidene fluoride-*co*-hexafluoropropylene) (PVDF-HFP) has a dielectric constant of ∼11, providing charge screening effects. Hence, this highly polar PVDF-HFP material has been employed as a matrix for solid polymer electrolytes (SPEs). In this study, the phase behavior of binary PVDF-HFP solutions was analyzed using the Flory–Huggins theory, in which ethylene carbonate, propylene carbonate, dimethyl carbonate, γ-butyrolactone, and acetone were employed as model solvents. In particular, for the binary PVDF-HFP/acetone system, the solid–liquid and liquid–liquid phase transitions were qualitatively described. Then, the phase diagram for ternary acetone/PVDF-HFP/polyphenolate systems was constructed, in which the binodal, spinodal, tie-line, and critical point were included. Finally, when a polyelectrolyte lithium polyphenolate was mixed with the PVDF-HFP matrix, it formed a single-ion conductor with a Li^+^ transference number of 0.8 at 23 °C. In the case of ionic conductivity, it was ∼10^−5^ S cm^−1^ in solid state and ∼10^−4^ S cm^−1^ in gel state, respectively.

## Abstract 2

A fluoropolymer poly(vinylidene fluoride-*co*-hexafluoropropylene) (PVDF-HFP) has a dielectric constant of ∼11, providing charge screening effects.

## Graphical abstract

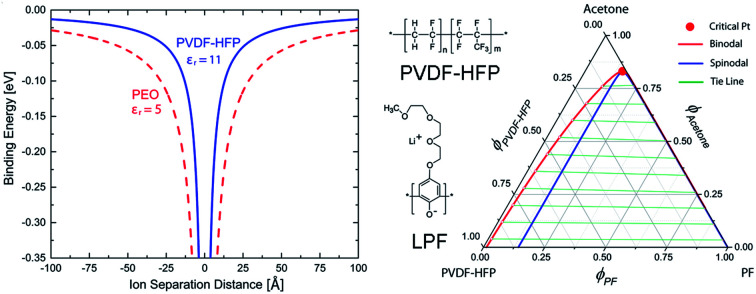

## Introduction

Poly(vinylidene fluoride-co-hexafluoropropylene) (PVDF-HFP) has been a benchmark matrix for solid-state polymer electrolytes due to its superior dielectric constant (*ε*_r_ ≈ 11), low glass transition temperature, high mechanical strength, and electrochemical stability.^1–6^ Specifically, the high *ε*_r_ affords a small binding energy with salt ions leading to an effective dissociation of charged particles, whereas the low *T*_g_ allows the polymer segmental motion (to be active) in its amorphous regions. These characteristics provide a pathway for an enhanced ionic conductivity depending on both charge concentration and mobility. Basically, all the properties of the fluoropolymer PVDF-HFP are largely governed by its chemical structure in the copolymer backbone. If the HFP content in the VDF/HFP units is less than 15–19 mol%, the copolymer is semicrystalline with thermoplastic properties, desirable for solid-state polymer electrolyte applications.^4–7^ The polymer electrolytes are composed of a polymer matrix and salt (or ionic liquid or polyelectrolyte), in which both plasticizer and active/passive filler could be added for improving ionic conductivity and mechanical properties, respectively.^8–11^ Furthermore, instead of a single polymer matrix, two different polymers can be mixed together to form a blend. For example, PVDF-HFP has been blended with other molecules such as poly(ethylene oxide) (PEO), poly(methyl methacrylate) (PMMA), poly(vinyl acetate) (PVAc), poly(vinyl chloride) (PVC), thermoplastic polyurethane (TPU), poly(methyl methacrylate-co-acrylonitrile-co-lithium methacrylate) (PMAML), poly(ionic liquid), polysiloxane, and carboxymethyl cellulose (CMC).^8,12–26^ Here, it is notable that these polymer electrolytes have been developed for solid-state batteries (SSBs), which are one of the post-lithium-ion batteries (PLIBs) including sodium-ion batteries (SIBs), lithium–sulfur batteries (LSBs), and lithium–air batteries (LABs).^27–32^ Specifically, the perfluoropolyether-based block copolymer electrolyte was designed for ultra-stable SIBs.^33^ In addition, versatile new concepts such as flexible cross-linked network electrolytes, metal–organic framework (MOF)-based electrolytes, porous organic cage ionic conductors, an aligned liquid crystalline polymer combined with ionic liquids and salt, polymer-in-salt electrolytes, and dual-/single-ion conductors have been introduced for the next-generation PLIB applications.^34–38^

In this study, I investigated the single-ion conductor based on the polymer blend composed of PVDF-HFP and lithium polyphenolate (LPF), in which LPF is a polyelectrolyte providing Li^+^ ions for the PVDF-HFP matrix. Through this work, I tried to improve the existing PEO/LPF-based single conductor^39^ by replacing PEO with the high-performance PVDF-HFP matrix. This is because PEO has a low dielectric constant (*ε*_r_ ≈ 5) and too high crystallinity (∼70–80%), leading to a relatively low ionic conductivity.^40–42^ Hence, this work was focused on the analysis of PVDF-HFP solutions and then, the application of PVDF-HFP/LPF blends to the solid polymer electrolytes (SPEs). Firstly, the phase behavior of binary and ternary PVDF-HFP solutions was investigated, for which the Flory–Huggins lattice theory was employed.^43–48^ To date, most studies on the phase behavior of PVDF or PVDF-HFP solutions have been carried out through experiments.^49–54^ An exception for this state is that Chen and his coworkers simply calculated the phase diagram of PVDF/dimethylacetamide (DMAc)/H_2_O and described the PVDF-membrane formation *via* nonsolvent induced phase inversion.^50^ In contrast, Wang *et al.* compared in their experiments the phase behavior of two different fluoropolymer-based ternary systems, *i.e.*, PVDF/DMAc/H_2_O and poly(vinylidenedifluoride-co-chlorotrifluoroethylene) (PVDF-CTFE)/DMAc/H_2_O.^52^ Here, the former was more easily phase-separable than the latter. Then, Shi *et al.* studied the effect of additive (LiCl and glycerol) on the phase behavior of PVDF-HFP/*n*-methyl-2-pyrrolidone (NMP)/H_2_O and found that the additive may alter the morphology and structure of the resulting membrane through a facilitated phase separation.^53^ Recently, Wei and his coworkers studied the effect of LiCl on phase behavior of the PVDF-CTFE/DMAc/H_2_O system and found that LiCl addition promoted both solid–liquid and liquid–liquid phase transitions, which is in line with Shi *et al.*'s results.^54^ Therefore, considering the deficiency of theoretical calculation for the PVDF-HFP solution thermodynamics, this work seems to bridge the gap in this field by providing theoretical predictions about the phase behavior of PVDF-HFP solutions in terms of binodal, spinodal, tie line, and critical point.^43–48^ For this purpose, the Flory–Huggins interaction parameter (*χ*) was essential, which was calculated based on solubility parameter (*δ*).^55,56^ However, if *δ* is unknown, it could be estimated through a group contribution method.^57^ Finally, with the understanding of phase behavior of PVDF-HFP solutions, PVDF-HFP was blended with a polyelectrolyte lithium polyphenolate (LPF) in acetone for the SPE applications. The resulting SPE showed a Li^+^-transference number of ∼0.8 as a single-ion conductor minimizing a concentration gradient and cell polarization in rechargeable lithium batteries.^10,58,59^

## Materials and methods

### Materials

PVDF-HFP (*M*_*n*_ ≈ 120.0 kg mol^−1^, *M*_*w*_ ≈ 400.0 kg mol^−1^, and polydispersity index (PDI) = 3.3) was provided from Elf Atochem, which is composed of VDF : HFP with 88 : 12 by mole ratio (Kynar-FLEX® 2801). *P*-Toluenesulfonyl chloride, HEPES buffer solution, horseradish peroxidase II (HRP II), NaOH, MgSO_4_, SiO_2_, hydroquinone, and other solvents were purchased from Sigma-Aldrich and used as received.

### Methods

^1^H nuclear magnetic resonance (NMR) spectra were obtained using an NMR spectrometer (Bruker). Infrared (IR) absorption data were obtained from IR spectrometer (Bomem, MB 100-C15) at 4000–400 cm^−1^. Here, the KBr disc method was used for sample preparation. Thermal analysis was carried out by differential scanning calorimetery (DSC) (DuPont model 910 thermal analyzer) at a scanning rate of 10 °C min^−1^ under N_2_ with a flow rate of 50 mL min^−1^. Note that, in this study, the DSC data were reported based on the first heating curve. Thermogravimetric analysis (TGA) was performed using a TA instruments over a temperature range of 25–700 °C at a scanning rate of 10 °C min^−1^ under N_2_ with a flow rate of 50 mL min^−1^. Impedance data were obtained for estimating both the ionic conductivity (*σ*) and the transference number of Li^+^ ions (*t*_Li^+^_) by using a frequency response analyzer (FRA, Solartron SI 1260), for which a stainless steel (SUS) or Li symmetrical cell was used, respectively. Note that the performance of polymer electrolyte (*e.g.*, the reproducibility of ionic conductivity data) was evaluated through the 1^st^ order linear fitting as a function of composition, indicating that both *y*-intercept and slope have the standard errors of ∼10^−6^ S cm^−1^. Through this regression analysis, the data distribution could be estimated although there should be experimental uncertainties.

**Scheme 1:**
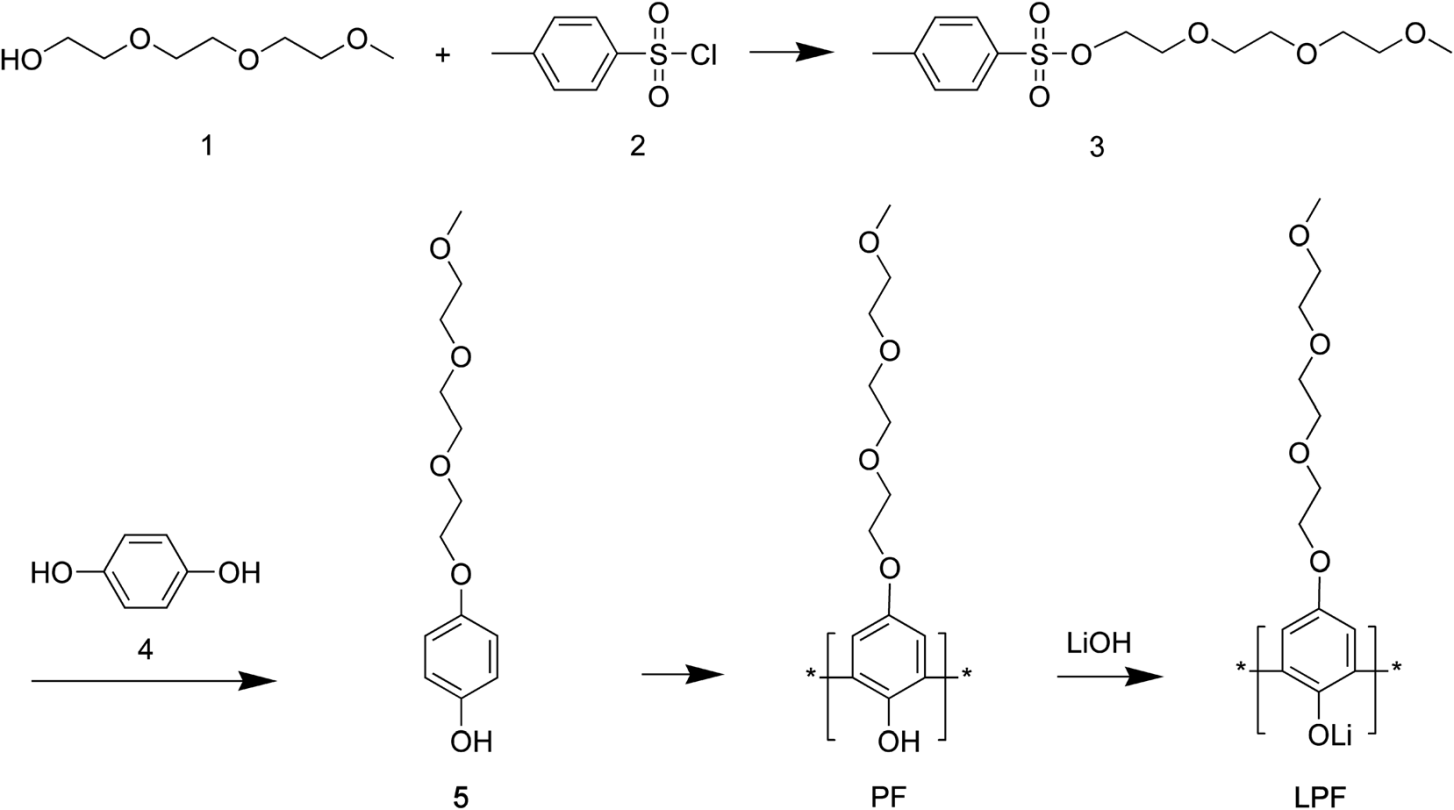
Synthesis of polyphenols: polyphenolate (PF) and lithium polyphenolate (LPF).

SPEs were prepared by dissolving LPF : PVDF-HFP (=1 : 1 to 1 : 5 by wt ratio) in acetone and cast it in a Teflon plate (see Scheme 1 for chemical structure of LPF). Then the cell with stainless steel (SUS)/SPE/SUS or Li/SPE/Li configuration was assembled and vacuum-sealed using a blue bag from Shield Pack, Inc. in a glove box under argon environment. Here, SUS/SPE/SUS is for estimating the bulk resistance and ionic conductivity of SPE, whereas Li/SPE/Li is for measuring Li^+^ ion's transference number (*t*_Li^+^_).

### Synthesis

#### Synthesis of (3)

NaOH (11.4 g, 0.285 mol) was dissolved in 60 mL H_2_O to make 5 M NaOH. Tri(ethylene glycol) monomethyl ether (1) (32.84 g, 0.2 mol) was dissolved in 50 mL THF (Scheme 1). Subsequently, two solutions were mixed together in 1000 mL flask under less than 5 °C. Then, *p*-toluenesulfonyl chloride (2) (36.2 g, 0.18 mol) in 50 mL of THF was dropwise added into the above solution and mixed. After about 2 hours, the solution's color was change into milky white, indicating the successful reaction. The solution was poured into 400 mL cold water. Using diethyl ether 50 mL, the synthesized chemical was extracted (×three times). It was washed several times and finally dried. The product was a transparent yellowish liquid.

#### Synthesis of (5)

Hydroquinone (4) (30.8 g, 279.71 mmol) and (3) (89 g, 279.75 mmol), KOH (39 g, 837 mmol) were mixed in 200 mL DMSO and then reacted for 12 hours at 23 °C. Then using dimethyl ether, HCl and chloroform, the product was extracted. It was washed using water, dried using MgSO_4_, and finally dried under vacuum at 23 °C.

#### Synthesis of PF

HRP II (24 mg) was dissolved in 55 mL HEPES buffer solution in 250 mL three-neck flask. Then (5) (8.83 g, 34.46 mmol) was dissolved in 25 mL 1,4-dioxane, and then this solution (5 in 1,4-dioxane) was added slowly into the above HRP-II/HEPES buffer solution. Then 30% H_2_O_2_ solution (1 mL) was added into this buffer solution, which was repeated for 5 times per 10 min under below 5 °C. Then solution's color was gradually changed to yellow. Then the reaction was allowed for additional ∼10–15 min under 23 °C and stirred overnight, resulting in dark brown color.

#### Synthesis of LPF

PF (4 g, 15.7 mmol) was dissolved in 20 mL 1,4-dioxane. LiOH (38 mg, 15.7 mmol) was dissolved in 10 mL water. Then two solutions were mixed together, resulting in a black colored solution. Then 1,4-dioxane and water was removed from the product using a rotary evaporator. Then the product was stored in vacuum overnight.

## Results and discussion

### Dielectric property of PVDF-HFP

**Fig. 1:**
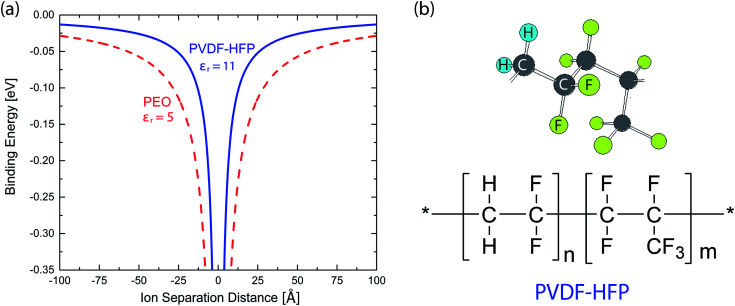
(a) Binding energy as a function of ion separation distance. Here, a monovalent cation is assumed to be at the origin, whereas a monovalent anion at the indicated distance. Potential wells were calculated for PVDF-HFP with *ε*_r_ ≈ 11 and PEO with *ε*_*r*_ ≈ 5. (b) Chemical structure of PVDF-HFP copolymer.

The electric potential energy (*U*_E_) between two charges (*q*_1_ and *q*_2_) in a dielectric is a function of the permittivity (*ε* = *ε*_0_*ε*_r_) of a material as well as charge-separation distance (*r*_12_).1
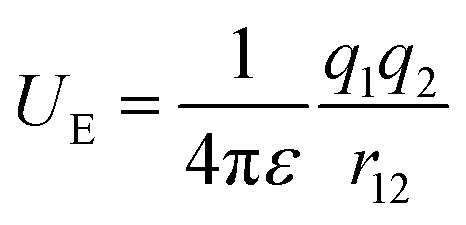
where *ε*_0_ and *ε*_r_ are the vacuum permittivity and a relative permittivity (or dielectric constant), respectively. Fig. 1a shows the coulombic potential well describing the binding energy between cation and anion when the dielectric constant of polymer is *ε*_r_ ≈ 11 for PVDF-HFP (its chemical structure in Fig. 1b) and *ε*_r_ ≈ 5 for PEO, respectively.^6,39,60^ According to eqn (1), when is high, the binding energy between cation and anion should be small because of the screening effect of a polarized medium on charges. Thus, PVDF-HFP displays a narrow coulombic potential well, whereas PEO exhibits a wide one. For example, when ion separation distance is 10 Å, the binding energy is 0.29 eV in PEO, whereas 0.13 eV in PVDF-HFP. This characteristic implies that the charge concentration for ionic conductivity will be higher in PVDF-HFP than in PEO. Thus, for developing SPEs, it is reasonable to replace PEO with PVDF-HFP or other high dielectric fluoropolymers, *e.g.*, PVDF-CTFE (*ε*_r_ ≈ 13), and poly(vinylidenefluoride-co-trifluoroethylene) (PVDF-TrFE) (*ε*_r_ ≈ 18).^3–6^

### Phase behavior of binary PVDF-HFP solutions

The Flory–Huggins theory can describe the phase behavior of polymer solutions, for which the two parameters such as interaction parameter (*χ*) and the relative molar volume of a polymer (*r*_2_) should be provided. In the case of solvent, *r*_1_ = 1. The molar Gibbs energy of mixing (Δ*G*_mix_) for a binary polymer solution is expressed as follows,^45,61^2
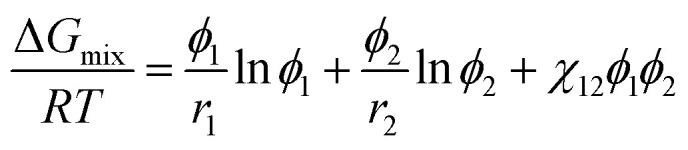
where *R*, *T*, and *ϕ*_*i*_ are the gas constant, temperature (K), and the volume fraction of component *i* (=1 for solvent and 2 for polymer). Here, *χ*_*ij*_ could be estimated from the solubility parameter information as follows,3
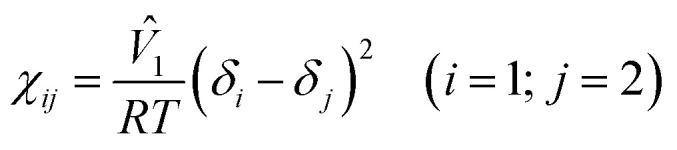
where *V̂*_1_ and *δ*_*i*_ are a molar volume of solvent and a solubility parameter of component *i*. Here, the *χ* parameter is inversely proportional to temperature. Then, the equilibrium condition of chemical potentials, Δ*μ*_*i*_ (= ∂Δ*G*_mix_/∂*n*_*i*_), is expressed as follows,

4 Δ*μ*^α^_*i*_ = Δ*μ*^β^_*i*_ (*i* = 1, 2)

where α and β indicate two different phases at equilibrium. Using eqn (4), the upper critical solution temperature (UCST) phase behavior could be predicted. Furthermore, the melting point of a binary polymer solution could be described as follows,5
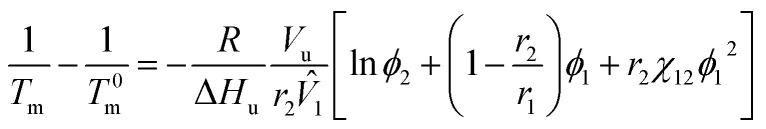
where *T*_m_ and *T*^0^_m_ are the temperatures of a binary solution and a pure polymer, respectively. Δ*H*_u_ is the enthalpy of polymer's structural unit (when crystallinity is 100%), and *V*_u_ is the volume of polymer's structural unit. In general, the chain length of polymer is much larger than that of solvent (*i.e.*, *r*_2_ ≫ *r*_1_ = 1). Thus, the eqn (5) could be reduced as follows,6
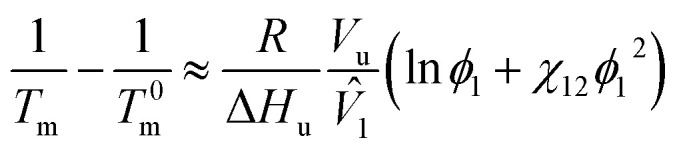
which is called Nish–Wang equation.^62^

**Fig. 2:**
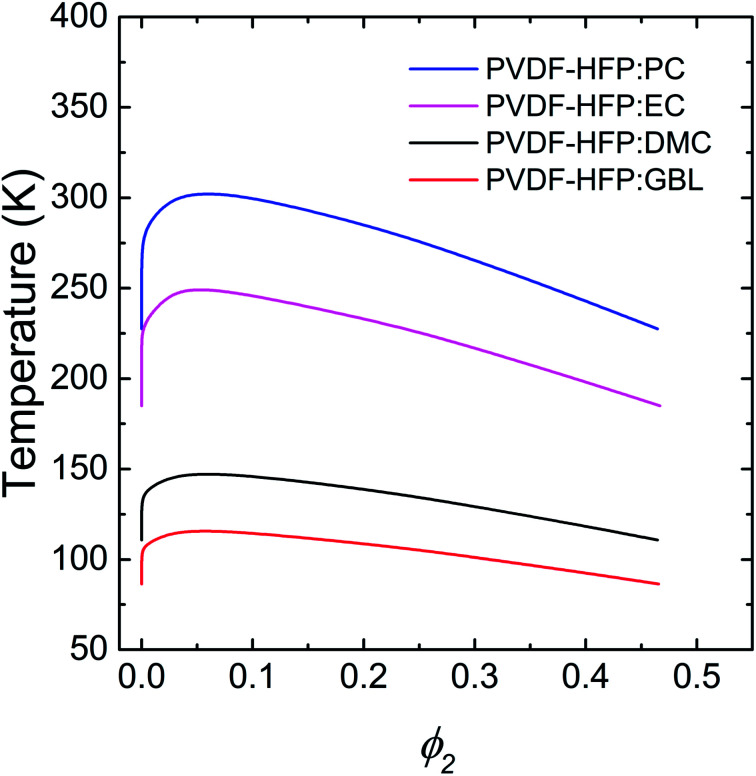
The UCST phase behavior of binary PVDF-HFP solutions, which was calculated based on the Flory–Huggins model. Here, PC, EC, DMC, and GBL were used as a model solvent or plasticizer. PVDF-HFP has *M*_*n*_ = 40.0 kg mol^−1^ and *δ* = 11.3 (cal cm^−3^)^1/2^.

Specifically, propylene carbonate (PC), ethylene carbonate (EC), dimethyl carbonate (DMC), and γ-butyrolactone (GBL) have been used as solvent (or plasticizer) for polymer electrolytes.^62–64^ Thus, using these solvents, the UCST phase behavior of PVDF-HFP solutions was calculated. As a first step, the model polymer (PVDF-HFP) was assumed to have a moderate molecular weight *M*_*n*_ = 40.0 kg mol^−1^ with *δ*_2_ = 11.3 (cal cm^−3^)^1/2^.^63^ Then, the properties of solvents were summarized in Table 1.^64^ According to the Flory–Huggins model, the PVDF-HFP solutions exhibited a better miscibility with the sequence of GBL > DMC > EC > PC, which was largely governed by *χ*_12_ parameters (Table 1). Note that smaller *χ*_12_, more miscible each other. In Fig. 2, the critical point (*ϕ*^c^_2_,*T*_c_) is (0.06, 302.96) for PVDF-HFP/PC, (0.05, 249.56) for PVDF-HFP/EC, (0.06, 147.56) for PVDF-HFP/DMC, and (0.06, 115.91) for PVDF-HFP/GBL, respectively. Here, it is notable that the Flory–Huggins model can capture ‘qualitatively’ the trend of UCST phase behaviors of binary polymer solutions.

**Table 1:** Solubility parameter (*δ*_1_), molecular weight (MW), density (*d*), molar volume (*V̂*_1_), relative molar volume (*r*_2_), and interaction parameter (*χ*_12_) for PVDF-HFP/solvent systems. Here, PVDF-HFP as a model system has *δ*_2_ = 11.3 (cal cm^−3^)^1/2^, *d* = 1.77 g cm^−3^, *M*_*n*_ = 40.0 kg mol^−1^, and molar volume = 22 600 cm^3^ mol^−1^

Solvent	*δ*_1_ (MPa^1/2^)	*δ*_1_ (cal cm^−3^)^1/2^	MW (g mol^−1^)	*d* (g cm^−3^)	*V̂*_1_ (cm^3^ mol^−1^)	*r*_2_ (−)	*χ*_12_ (−)
EC	30.1	14.7	88.06	1.3214	66.64	339	138.70 K/*T*
PC	27.2	13.3	102.09	1.2047	84.74	267	170.59 K/*T*
GBL	25.8	12.6	86.06	1.1284	76.29	296	64.89 K/*T*
DMC	20.3	9.9	90.08	1.0697	84.21	268	83.07 K/*T*
Ace	20.3	9.9	58.08	0.7845	74.03	305	73.02 K/*T*

**Fig. 3:**
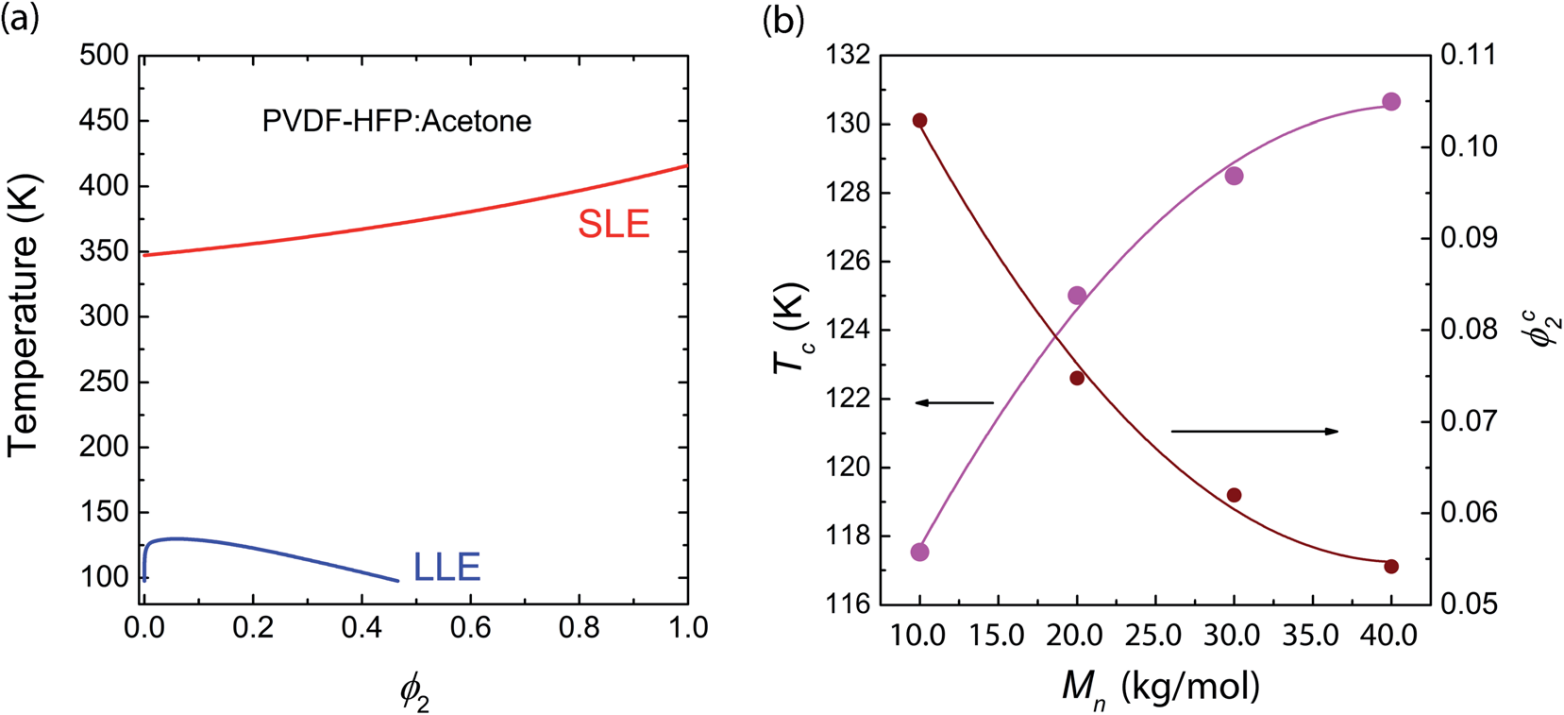
Phase behavior of PVDF-HFP/Acetone systems: (a) LLE from Flory–Huggins model, and SLE from Nish–Wang model. (b) Critical points (*ϕ*^c^_2_,*T*_c_) as a function of*M*_*n*_. When the number average molecular weight (*M*_*n*_) is 10.0, 20.0, 30.0, and 40.0 kg mol^−1^, the relative molar volume (*r*_2_) is 76, 153, 209 and 305, respectively.

For the binary PVDF-HFP/acetone (Ace) system, not only liquid–liquid phase equilibria (LLE), but also solid–liquid phase equilibria (SLE) were calculated because the acetone was used as a processing solvent for fabricating a SPE in this study. Note that both Ace and DME have the same *δ*_1_ = 9.9 (cal cm^−3^)^1/2^, but they have different molecular weights and densities, distinguishing the phase behavior. Hence, the LLE curve for PVDF-HFP/Ace was slightly different from that of PVDF-HFP/DMC. Furthermore, using eqn (6), *i.e.*, Nish–Wang model, the SLE curve was calculated as shown in Fig. 3a. Here, the parameters used are Δ*H*_u_ = 104.7 J g^−1^ × 202 g mol^−1^ = 21 148.4 J mol^−1^, *V*_u_ = 114 cm^3^ mol^−1^, *V̂*_1_ = 74.03 cm^3^ mol^−1^, *T*^0^_m_ = 143 + 273 = 416 K, and *R* = 8.314 J mol^−1^ K^−1^. Note that PVDF-HFP copolymer has the unit molecular weight of 202 g mol^−1^ and an enthalpy of fusion, *ca.* 104.7 J g^−1^.^65,66^

The phase behavior in Fig. 3a suggests that PVDF-HFP may be crystallized out before liquid–liquid (L–L) phase separation if kinetics is sufficiently slow. Furthermore, the critical points (*ϕ*^c^_2_,*T*_c_) were calculated for various molecular weights (or chain lengths) of PVDF-HFP (Fig. 3b). The critical point (*ϕ*^c^_2_,*T*_c_) is (0.10, 117.53) for 10.0 kg mol^−1^, (0.08, 125.01) for 20.0 kg mol^−1^, (0.06, 128.50) for 30.0 kg mol^−1^, and (0.05, 130.65) for 40.0 kg mol^−1^, respectively. As shown in Fig. 3b, the slope of curves decreases with increasing *M*_*n*_, indicating a saturation behavior.

### Phase behavior of ternary PVDF-HFP solutions

The Flory–Huggins model (eqn (2)) could be extended for describing the phase behavior of ternary polymer solutions with components, *i* = 1, 2, 3.^43,47^7
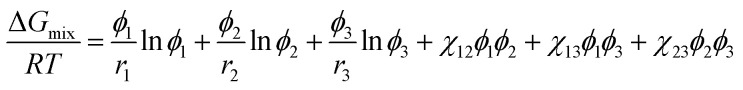
where *χ*_*ij*_ = *V̂*_1_/*RT*(*δ*_*i*_ − *δ*_*j*_)^2^ with *i* or *j* = 1, 2, 3 from eqn (2). In this work, the components, 1, 2, and 3 correspond to acetone, PVDF-HFP, and PF, respectively. Importantly, Δ*G*_mix_ in the eqn (7) is expressed ‘per lattice site’.^61^ However, if one converts Δ*G*_mix_ into Δ*G*^sys^_mix_ = Δ*G*_mix_/(*n*_1_*r*_1_ + *n*_2_*r*_2_ + *n*_2_*r*_2_) ‘per the system’ by multiplying (*n*_1_*r*_1_ + *n*_2_*r*_2_ + *n*_2_*r*_2_) in both right- and left-hand sides, the Flory–Huggins model could be expressed as follows,^47,61^8
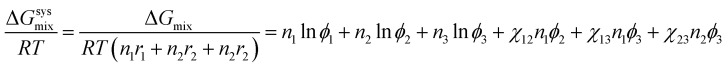
where *n*_*i*_is the number of moles of component *i*. Now for calculating the phase boundary for ternary systems, the chemical potential (Δ*μ*_*i*_) of component *i* should be expressed by differentiating the free energy of mixing in eqn (8) per the system.^46,48^9
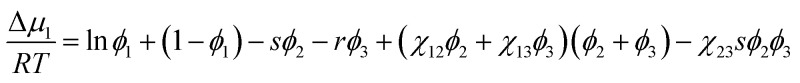
10
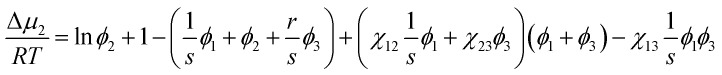
11
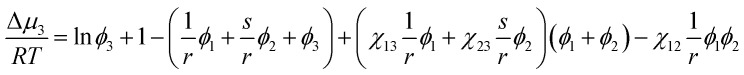
where *s* = *v*_1_/*v*_2_,*r* = *v*_1_/*v*_3_, and *s*/*r* = *v*_3_/*v*_2_. Here, *v*_*i*_ is molar volume of component *i*. The binodal curve could be estimated for ternary systems by extending eqn (2) as below.

12 Δ*μ*^α^_*i*_ = Δ*μ*^β^_*i*_ (*i* = 1, 2, 3)

Furthermore, the spinodal curve and the critical point could be calculated according to my previous work.^67^

For constructing the phase diagram of ternary Ace/PVDF-HFP/PF system, the eqn (12) was employed, enabling the calculation of the phase boundaries. Here, it is noteworthy that the Flory–Huggins theory can consider only van-der-Waals forces in terms of the *χ* interaction parameter. Thus, it was assumed that PF is a non-ionizable molecule as a model molecule. However, the density and solubility parameter for PF are unknown. Hence, they were estimated through the group contribution method as summarized in Tables 2 and 3.^57^ Accordingly, the density of PF is 1.74 g cm^−3^ (=∑*M*_*i*_/∑*V*_w*i*_ = 257.92/148.35), where *M*_*i*_ (g mol^−1^) and *V*_w*i*_ (cm^3^ mol^−1^) are the mass and van der Waals volume of structural group *i*, respectively. The molar volume is 987 cm^3^ mol^−1^, which was estimated from the relation of *M*_*n*_/*d* = 1717/1.74. The unit-molar volume (*V*_u_) is 193 cm^3^ mol^−1^ from unit molecular weight divided by density like MW_u_/*d* = 336/1.74. Lastly, the solubility parameter of PF was estimated to be 16.6 (cal cm^−3^)^1/2^ = 34.1 MPa^1/2^ by using the Hoftyzer–Van Krevelen method as below.13
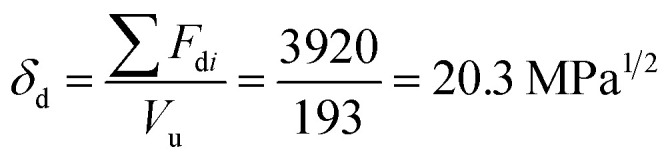
14
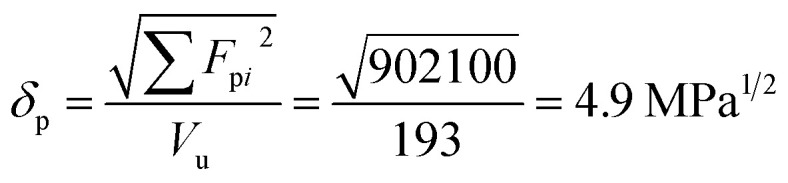
15
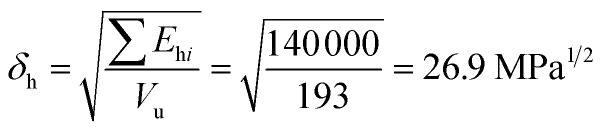
where *F*_d*i*_ and *F*_p*i*_ are force contributed from dispersion and polar components, respectively. *E*_h*i*_ is an energy contributed from hydrogen bonding component. Then, the solubility parameter was finally calculated from the relation, *δ*
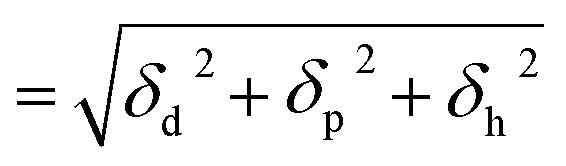

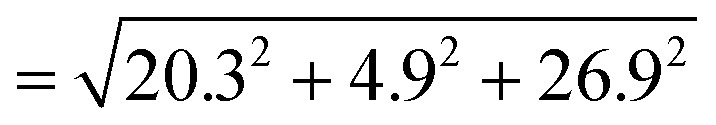
 = 34.1 MPa^1/2^was converted to 16.6 (cal cm^−3^)^1/2^ for estimating the *χ* interaction parameter.

**Table 2:** Group increments of mass and van der Waals volume for polyphenolate (PF)

Structural group	Number of group	*M*_*i*_ (g mol^−1^)	*M*_*i*_(cm^3^ mol^−1^)
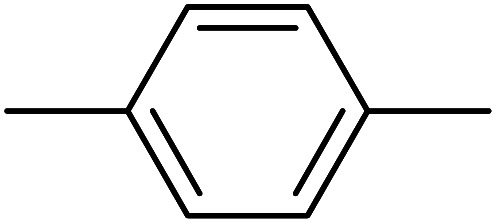	1	76.09	43.3
–O–	4	16	5.5
–OH–	1	17	8.0
–CH_2_–	6	14.3	10.23
–CH_3_	1	15.03	13.67

**Table 3:** Solubility parameter component group contributions (Hoftyzer–Van Krevelen method) for polyphenolate (PF)

Structural group	Number of group	*F*_d*i*_ (MJ m^−3^)^1/2^ mol^−1^	*F*_p*i*_ (MJ m^−3^)^1/2^ mol^−1^	*E*_h*i*_ J mol^−1^
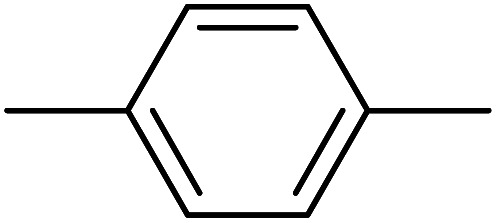	1	1270	110	0
–O–	4	100	400	30 000
–OH–	1	210	500	20 000
–CH_2_–	6	270	0	0
–CH_3_	1	420	0	0

**Fig. 4:**
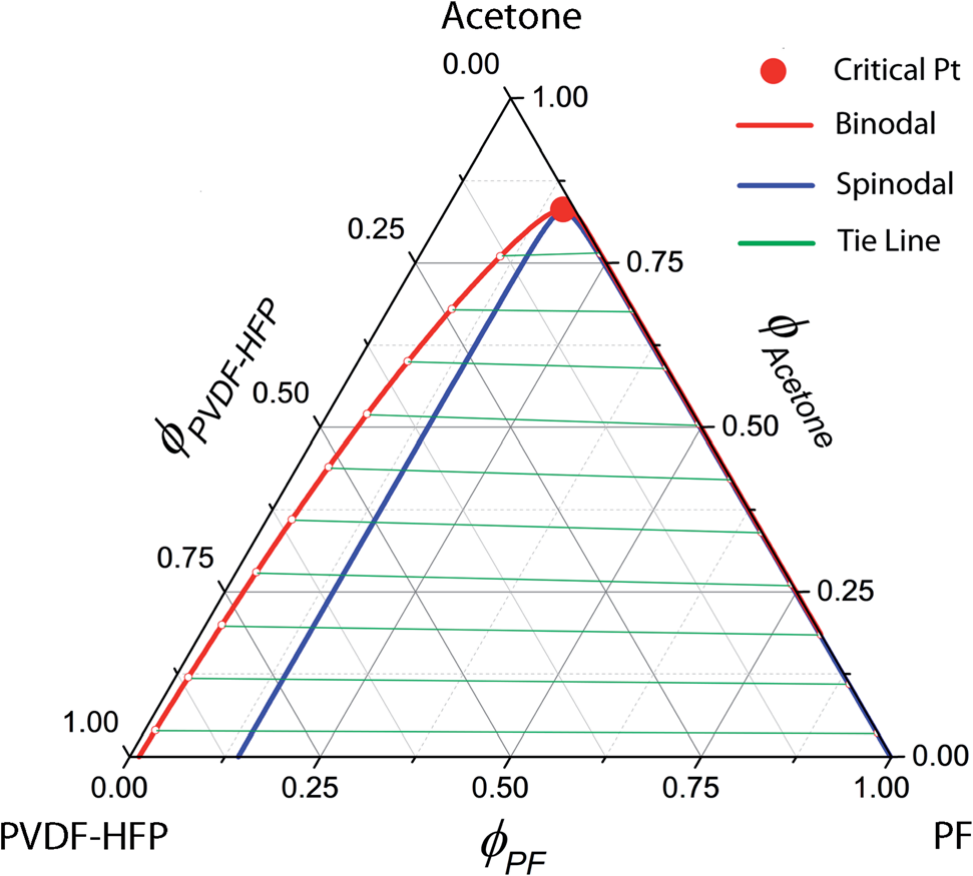
Phase diagram for the ternary Ace/PVDF-HFP/PF system. *T* = 298 K; *χ*_12_ = 0.25;*χ*_13_ = 5.61;*χ*_23_ = 3.51; *s* = 0.001091; *r* = 0.075022. PVDF-HFP's properties: *M*_*n*_ = 120.0 kg mol; *d* = 1.77 g cm^3^; molar volume = 67796.61 cm^3^ mol; *δ* = 11.3 (cal cm^−3^)^1/2^. PF's properties: *M*_*n*_ = 1.7 kg mol; *d* = 1.74 g cm^3^; molar volume = 986.78 cm^3^ mol; *δ* = 16.6 (cal cm^−3^)^1/2^. Acetone's properties: Molar volume = 74.03 cm^3^ mol; *δ* = 9.9 (cal cm^−3^)^1/2^.

Fig. 4 shows the phase diagrams for a ternary Ace/PVDF-HFP/PF system, in which the components 1, 2, and 3 correspond to acetone, PVDF-HFP, and PF, respectively. Here, the physical parameters are *χ*_12_ = 0.25,*χ*_13_ = 5.61,*χ*_23_ = 3.51, *s* = *v*_1_/*v*_2_ = 0.001091, and *r* = *v*_1_/*v*_3_ = 0.075022 at *T* = 298 K (Table 4). Resultantly, the phase diagram exhibited the critical point at (*ϕ*^c^_1_, *ϕ*^c^_2_, *ϕ*^c^_3_) = (0.83152, 0.01541, 0.15308), indicating that the phase-separation regions are very large. Specifically, when *ϕ*_1_ ≈ 0, the metastable region is from *ϕ*_3_ ≈ 0.01223 to 0.14265, whereas the unstable region is from *ϕ*_3_ ≈ 0.14265 to 1. Here, it is noteworthy that the nucleation-growth is undergone in a metastable region, whereas the spinodal decomposition proceeds in an unstable region. Hence, the dominant phase-separation process is through the spinodal decomposition in most compositions. Furthermore, two polymers are usually immiscible because of no entropic gain, *i.e.*, Δ*G*_mix_ = Δ*H*_mix_ − *T*Δ*S*_mix_ ≈ Δ*H*_mix_ ≥ 0. In particular, for the PVDF-HFP/PF blend, *χ*_23_ = 3.51 at *T* = 298 K, whereas the critical interaction parameter (*χ*^c^_23_) is 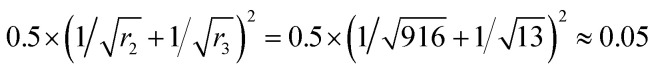.^62^ Note that if only*χ*_23_ < *χ*^c^_23_, two polymers are miscible. However, currently, *χ*_23_ = 3.51 > *χ*^c^_23_ = 0.05, indicating PVDF-HFP and PF are immiscible.

**Table 4:** Physical parameters for calculating the phase diagrams of ternary Ace/PVDF-HFP/PF systems when *T* = 298 K,*χ*_12_ = 0.25,*χ*_13_ = 5.61,*χ*_23_ = 3.51, *s* = 0.001091, and *r* = 0.075022. Here, MW = molecular weight; *d* = density; *v*_*i*_ = molar volume; and *δ*_*i*_ = solubility parameter

Materials	Component *i*	MW (g mol^−1^)	*d* (g cm^−3^)	*v*_*i*_ (cm^3^ mol^−1^)	*δ*_*i*_ (MPa^1/2^)	*δ*_*i*_ (cal cm^−3^)^1/2^
Ace	1	58.08	0.7845	74.03	20.3	9.9
PVDF-HFP	2	120 000^a^	1.77	67 796.61	23.2	11.3
PF	3	1,717^a^	1.74	986.78	34.1	16.6

a Number average molecular weight (*M*_*n*_).

Interestingly, the phase behavior of the Ace/PVDF-HFP/PF system in Fig. 4 is very similar to that of the solvent/polymer/nonsolvent system (*e.g.*, NMP/PVDF-HFP/H_2_O).^53,54^ Hence, just like nonsolvent induced phase separation (NIPS), it is expected that PF-induced phase separation takes place in the Ace/PVDF-HFP/PF system because PVDF-HFP and PF are immiscible. Importantly, according to Shi *et al.*, the addition of salts (*e.g.*, LiCl) into this solvent/polymer/nonsolvent system induced the binodal to shift further towards the polymer–solvent axis (*i.e.*, an enlarged phase-separation probability).^53^ At this moment, it is important to remind that the Flory–Huggins theory cannot deal with coulombic interactions. However, if the ternary Ace/PVDF-HFP/LPF system (here, LPF is ionizable) is considered, the two phenomena are basically expected. One is that the ionization of LPF may increase the entropy of electrolyte system, and the other is that Li^+^ ions may have a coordination bonding (a weak transient crosslinking) with Lewis base (Florine) in PVDF-HFP, indicating the modified intra-/inter-molecular interactions (*i.e.*, enthalpy) among component molecules. Hence, based on the two thermodynamic (entropic and enthalpic) effects, the phase-separation boundaries should be shifted for adjusting a new equilibrium point.^53,54^

### Single Li-ion conducting solid polymer electrolyte: synthesis, properties and performance

**Fig. 5:**
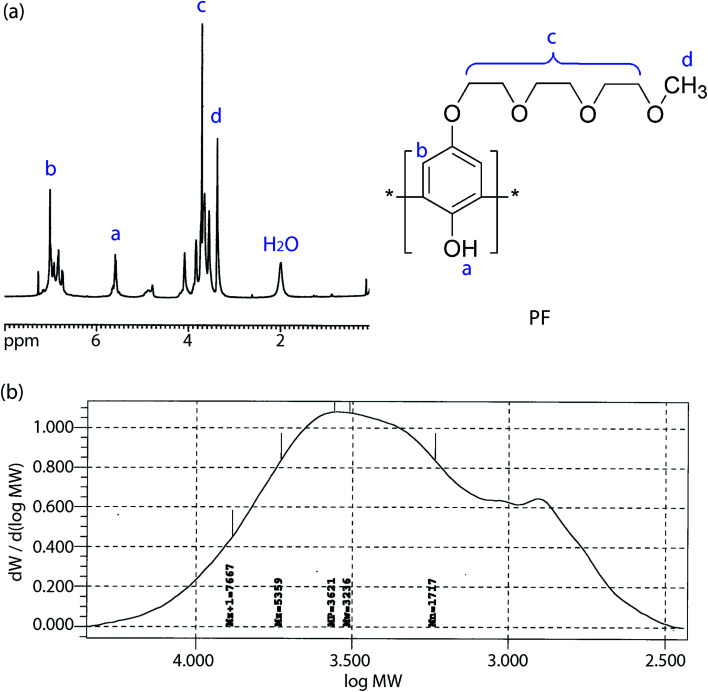
(a) ^1^H NMR spectra and chemical structure of PF. (b) GPC data for PF: *M*_*n*_ = 1.7 kg mol^−1^, *M*_*w*_ = 3.2 kg mol^−1^, and PDI ≈ 1.9.

**Fig. 6:**
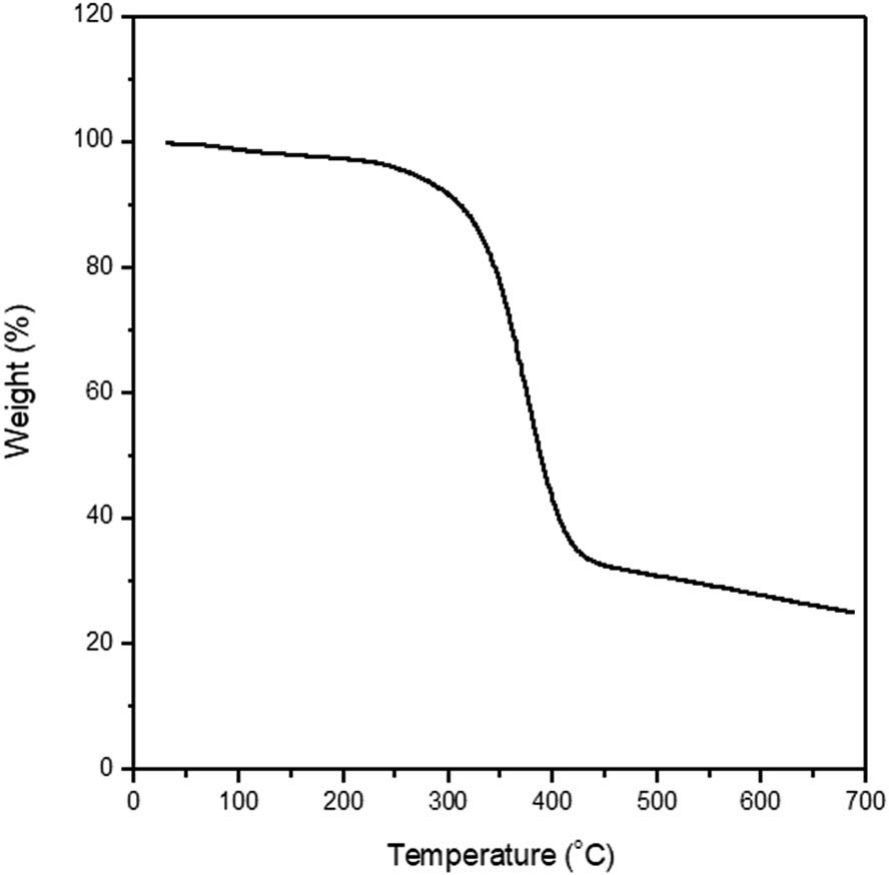
TGA data for polyphenolate (PF).

In the backdrop of aforementioned PVDF-HFP thermodynamics, LPF was synthesized and blended with PVDF-HFP using acetone (recall Scheme 1). Fig. 5a shows ^1^H NMR spectra for PF, *i.e.*, a precursor for LPF, in which ‘–H from benzene, –OH, –CH_2_–, and –CH_3_’ were observed at ∼7 ppm, ∼5.5 ppm, ∼4–3.5 ppm, and ∼3.7 ppm, respectively. The molecular weights of PF are about *M*_*n*_ ≈ 1.7 kg mol^−1^ (equivalent to ∼7 structural units) and *M*_*w*_ ≈ 3.2 kg mol^−1^, indicating that PDI ≈ 1.9. Here, it is noticeable that the spatial size of PF is close to oligomer, suggesting a partial increase of (*ϕ*_3_/*r*_3_) ln *ϕ*_3_ in eqn (8) by reducing the relative molar volume (*r*_3_) of PF. In addition, Fig. 6 shows the TGA data for the synthesized PF, displaying the major thermal decomposition at ∼300–400 °C.

**Fig. 7:**
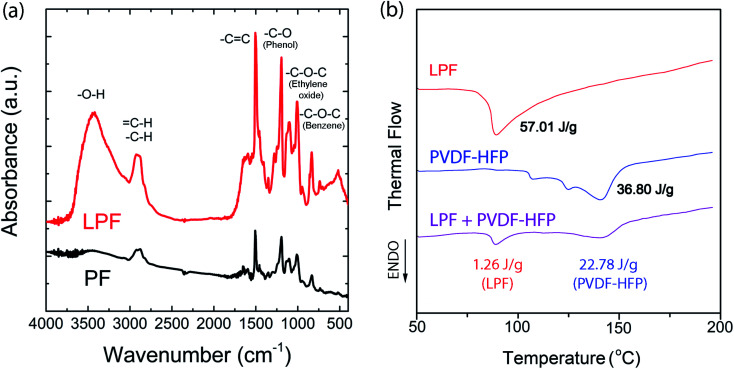
(a) IR spectra for both PF and LPF. (b) DSC thermograms for LPF, PVDF-HFP, and LPF : PVDF-HFP = 1 : 1.7 (wt ratio) mixture.

As a next step, I examined the infrared (IR) spectra for PF and LPF, respectively. As shown in Fig. 7a, –O–H stretching at 3438 cm^−1^, –C–H stretching at 2881 cm^−1^, –C=C stretching at 1610 cm^−1^ and 1500 cm^−1^, –C–O (phenol) stretching at 1192 cm^−1^, –C–O–C (ethylene oxide) stretching at 1106 cm^−1^, and (Bz)–C–O–C stretching at 1004 cm^−1^ were observed. Interestingly, based on the IR spectra, LPF and PF show a partial difference in the relative intensity (not position) of peaks. Notably, the –O–H stretching was observed at 3438 cm^−1^ for a hygroscopic LPF sample also, suggesting the H_2_O absorption during FT-IR measurement in air. However, it is notable that in the case of electrical measurement, the device was fabricated in a glove box under argon environment, not in air.

Fig. 7b shows the DSC thermogram for LPF, PVDF-HFP, and the polymer blend LPF : PVDF-HFP = 1 : 1.7, which was recorded during the first heating scans at 10 °C min^−1^. The melting points (*T*_m_) of the pure components (LPF and PVDF-HFP) were observed at 87 °C and 142 °C, respectively. In the case of LPF : PVDF-HFP = 1 : 1.7, the blend exhibited *T*_m_ at the same temperature of each components, indicating that PVDF-HFP and LPF are basically immiscible as expected from most two polymers. As shown in Fig. 7b, when blended, the enthalpy was reduced from 57.01 J g^−1^ to 1.26 J g^−1^ for LPF, whereas from 38.08 J g^−1^ to 22.78 J g^−1^ for PVDF-HFP, indicating the diminished crystallinity (98% reduction for LPF and 38% for PVDF-HFP). Here, if one compares PVDF-HFP and LPF, the crystalline region of PVDF-HFP could survive more than that of LPF when blended each other. Finally, in the case of PVDF-HFP, its ideal enthalpy of melting is 104 J g^−1^.^68^ Hence, the crystallinity of pure PVDF-HFP is *ca.* 35.39% = (36.80/104) × 100, whereas that of PVDF-HFP in the LPF/PVDF-HFP mixture is *ca.* 21.90% = (22.78/104) × 100.

**Fig. 8:**
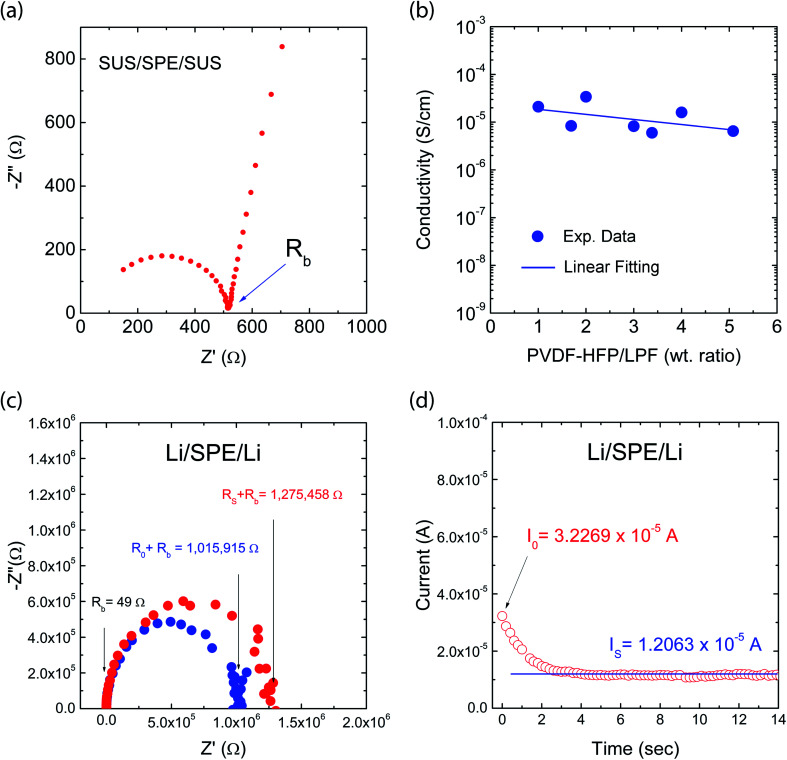
Ionic conductivity: (a) Example of Nyquist plot. (b) Ionic conductivity as a function of composition for a single Li-ion conducting solid polymer electrolyte based on PVDF-HFP and LPF mixtures. Li transference number: (c) The AC impedance spectra for the cell before and after DC polarization at 23 °C. (d) The current response for Li/SPE/Li cell as a function of time during DC polarization under a constant potential of 10 mV at 23 °C.

Fig. 8a shows a typical Nyquist plot of the impedance data for the PVDF-HFP/LPF system at 23 °C, displaying a bulk resistance (*R*_b_), for which the symmetric cell with a SUS/SPE/SSU structure was fabricated. Here, the ionic conductivity (*σ*) could be estimated from the relation of *l*/(*R*_b_*A*), where *l* and *A* are thickness and area of a film, respectively. For example, when *R*_b_ = 514.25 Ω, *l* = 172 μm, and *A* = 4 cm^2^, *σ* would be 8.4 × 10^−6^ S cm^−1^. In the same way, the resulting ionic conductivity as a function of composition is displayed in Fig. 8b. In this range of 1 ≤ wt_PVDF-HFP_/wt_LPF_ ≤ 5, the average ionic conductivity is *σ* = 1.4 × 10^−5^ S cm^−1^ with the maximum *σ* = 3.4 × 10^−5^ S cm^−1^ at wt_PVDF-HFP_/wt_LPF_ = 2 and the minimum *σ* = 6.5 × 10^−6^ S cm^−1^ at wt_PVDF-HFP_/wt_LPF_ = 5. Although there were partial fluctuations in data, the overall trend indicated that the ionic conductivity was enhanced with increasing the LPF amounts in the SPEs. Furthermore, when the experimental data were fitted linearly, the result was *y* = (2.45 × 10^−5^) + (−3.56 × 10^−6^)*x* with the standard errors (*y*-intercept: 8.97 × 10^−6^ S cm^−1^ and slope: 2.83 × 10^−6^ S cm^−1^), in which *y* and *x* denote ionic conductivity and weight fraction (wt_PVDF-HFP_/wt_LPF_), respectively. On the other hand, it is notable that the ionic conductivity for PEO/LPF was reported to be ∼10^6^ S cm^−1^ at 23 °C and ∼10^5^ S cm^−1^ at 100 °C,^39^ indicating that PVDF-HFP/LPF is superior to PEO/LPF as a polymer electrolyte system. Furthermore, the Li^+^ ion's transference number (*t*_Li^+^_) for the PVDF-HFP/LPF system was estimated based on the below relation,^10,69^16
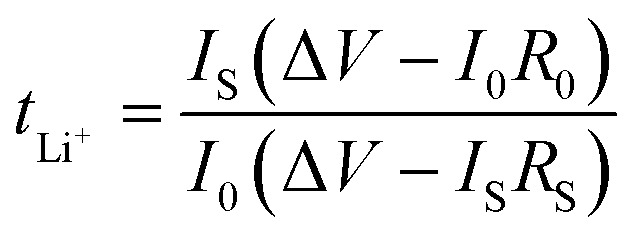
where *I*_0_ and *I*_S_ are the initial and steady-state currents under the DC polarization voltage (Δ*V* = 10 mV), *R*_0_ and *R*_S_ are the initial and steady-state interfacial resistance by the AC impedance method before and after DC polarization. For the PVDF-HFP/LPF system, the AC impedance spectra for the cell were measured before and after DC polarization as shown in Fig. 7c. Here, during the DC polarization under a constant potential of 10 mV, the current response was measured for the Li/SPE/Li cell as a function of time as shown in Fig. 7d. Resultantly, *t*_Li^+^_ ≈ 0.8 was estimated using eqn (16) with the values of Δ*V* = 0.01 V, *I*_0_ = 3.2269 × 10^−5^ A, *I*_S_ = 1.2063 × 10^−5^ A, *R*_0_ = 1 015 915–49 = 1 015 866 Ω, and *R*_S_ = 1 275 458–49 = 1 275 409 Ω, which is a promising result as a single-ion conducting SPE. However, it is notable that *t*_Li^+^_ is only 0.8 below the ideal ∼1.0, indicating that 20% of electricity was transferred through anions. Hence, although minus charges were embedded in the oligomeric polyphenolate with PDI ∼ 1.9, some anions (relatively smaller molecules among polydisperse LPFs) may migrate under the electric field. This phenomenon suggests that for increasing *t*_Li^+^_, the minus charges should be well fixed on the macromolecular polyelectrolyte. More importantly, although many research groups have reported *t*_Li^+^_ at high temperature (*e.g.*, ∼60–90 °C),^70–74^ here I reported *t*_Li^+^_ at 23 °C. This observation suggests that the polar polymer PVDF-HFP is an effective matrix for ionic conductor applications.

Finally, although the research theme of mine was in the development of solid-state polymer electrolyte, I carried out the preliminary study about the filler and plasticizer effect on the ionic conductivity. Resultantly, when the inorganic filler SiO_2_ was added ∼25 wt% of LPF, the ionic conductivity (LPF : PVDF-HFP : SiO_2_ = 1 : 1.7 : 0.25) was improved up to ∼1.03 × 10^−4^ S cm^−1^ (compared to average 1.4 × 10^−5^ S cm^−1^ without filler). Furthermore, when a plasticizer (EC : PC = 1 : 1) was added into the above system like LPF : PVDF-HFP : SiO_2_:EC : PC = 1 : 1.7 : 0.25 : 0.5 : 0.5, the ionic conductivity was similarly ∼1.0–1.1 × 10^−4^ S cm^−1^ (see Table 5). This indicates that there is a trade-off relationship between the flexibility (increase of chain motion) and dilution (decrease of carrier concentrations per volume) of electrolyte system. Hence, the ionic conductivity was ∼10^−4^ S cm^−1^ in the case of the aforementioned gel polymer electrolyte (GPE).

**Table 5:** Ionic conductivity of each polymer electrolyte at 23 °C

	Polymer electrolyte (weight ratio)			
	LPF : PEO = 1 : 3.57^a^	LPF : PVDF-HFP = 1 : 1.7	LPF : PVDF-HFP : SiO_2_ = 1 : 1.7 : 0.25	LPF : PVDF-HFP : SiO_2_ : EC : PC = 1 : 1.7 : 0.25 : 0.5 : 0.5
*σ* (S cm^−1^)	∼10^−6^	∼3.4 × 10^−5^	∼1.0 × 10^−4^	∼1.1 × 10^−4^

a LPF : PEO = 1 : 3.57 (wt ratio) indicates that the 20 repeat units of PEO per repeat unit of LPF.^21^

## Conclusion

When the highly polar PVDF-HFP fluoropolymer was blended with a polyelectrolyte lithium polyphenolate (LPF) for single-ion conductor applications, the ionic conductivity was ∼10^−5^ S cm^−1^ in solid state and ∼10^−4^ S cm^−1^ in gel state. More importantly, this LPF/PVDF-HFP single-ion conductor displayed a lithium transference number of *ca.* 0.8 at 23 °C, indicating that 20% of charges were transported through the anions fixed in the oligomeric polydisperse polyphenolate. Finally, considering the limited thermodynamic studies on the phase behavior of PVDF-HFP solutions and blends, I believe this work should be a significant progress, providing the insight for the phase behavior of PVDF-HFP solutions and blends based on the classical Flory–Huggins lattice theory.

## Conflicts of interest

The authors declare no competing financial interest.

## Supplementary Material
